# Editorial: Actinobacteria in Special and Extreme Habitats: Diversity, Function Roles, and Environmental Adaptations

**DOI:** 10.3389/fmicb.2016.01415

**Published:** 2016-09-08

**Authors:** Sheng Qin, Wen-Jun Li, Syed G. Dastager, Wael N. Hozzein

**Affiliations:** ^1^The Key Laboratory of Biotechnology for Medicinal Plant of Jiangsu Province, Jiangsu Normal UniversityXuzhou, China; ^2^State Key Laboratory of Biocontrol and Guangdong Provincial Key Laboratory of Plant Resources, School of Life Sciences, Sun Yat-Sen UniversityGuangzhou, China; ^3^Council of Scientific and Industrial Research National Chemical Laboratory, National Chemical Laboratory Resource CenterPune, India; ^4^Zoology Department, College of Science, King Saud UniversityRiyadh, Saudi Arabia

**Keywords:** actinobacteria, special and extreme environments, diversity, omics technologies, activities, environmental adaptation

The phylum *Actinobacteria* composes a diverse group of Gram-positive bacteria with high G + C content, which are abundant in soils and present in various special and extreme habitats. Actinobacteria have made a significant contribution to the health and well-being of people throughout the world (Demain and Sanchez, 2009). However, the increasing emergence of new diseases and pathogens, and the antibiotic resistance question in recent years have caused a resurgence of interest in finding new biologically active compounds for drug discovery. Thus, previously unexplored ecological niches and regions in the world have been pursued as sources of novel actinobacteria and antibiotics and other useful biologically active agents (Tiwari and Gupta, 2012). With Prof. William C. Campbell and Satoshi Omura winning the Nobel Prize in Physiology or Medicine in 2015 for their discovery of Avermectin, the discovery of new antibiotics from actinobacteria is expected to enter a new golden age.

Actinobacteria have been isolated from diverse ecosystems, including alkaline saline soil, marine sponges, and deep sea sediments, hot springs, guts, and medicinal plants. They have broad applications potential in agriculture and environmental protection apart from antibiotic production due to their diverse ecological functions. During the last few decades, actinobacterial resource research has focused on special habitats and extreme environments; however, due to the limitations of isolation and cultivation methods, our knowledge of the diversity and ecology of extremophilic actinobacteria is at best fragmentary (Bull, 2011). Recent advances in microbial cultivation, next generation sequencing (NGS) technologies and -omics (metagenomics, metaproteomics etc) methods have greatly contributed to the rapid advancement of our understanding of actinobacterial diversity from special and extreme habitats (Qin et al., 2012; Hamedi et al., 2013; Orsi et al., 2016). Still, the physiological functions of actinobacteria and their environmental interactions await further investigation.

We proposed this research topic to highlight the current advances and knowledge related to actinobacteria from extreme environments. In this Research Topic e-book “Actinobacteria in special and extreme habitats: diversity, function roles and environmental adaptations” we collected 17 articles, including 4 reviews and 13 original articles that focus on actinobacterial species diversity from different special and extreme habitats, as well as the bioactive secondary metabolites, functional genes and potential ecological functions of actinobacteria. We are grateful to all authors who have submitted contributions to this research topic.

Actinobacteria in extreme habitats represent not only extensive taxonomic diversity, but also high genetic diversity of their biosynthetic pathways for synthesizing novel biological compounds. Mohammadipanah and Wink review the diversity and biotechnological potential of actinobacteria from arid and desert habitats. The article by Shivlata and Satyanarayana also reviews the taxonomic diversity of thermophilic and alkaliphilic actinobacteria, and discusses their potential applications in industry, agriculture and biotechnology. Sun H. M. et al. provide an example of physiological characteristics of a predominant actinobacterial group, found in their survey of highly diverse culturable but rare actinobacteria in desert soil crusts. Interestingly, the article by Riquelme et al. explores the actinobacteria in volcanic caves using culture-dependent and culture-independent methods; the results help fill in the gaps in our knowledge of actinobacterial diversity and their potential ecological roles in the volcanic cave ecosystems. Two articles by Yang et al. and Tang et al. use 16S rRNA gene clone library construction to describe the diversity of actinobacteria in the ecologically sensitive Yanshan Mountains zone and in cold springs sediments in China; they found that biogeographical isolation and biogeochemical factors might be major factors influencing actinobacterial distribution. Many articles focusing on marine actinobacteria are also present. Ser et al. and Tan et al. report bioactive *Streptomyces* species from coastal mangrove soil in Malaysia and their antioxidative metabolites. Marine actinobacteria, particularly coral and sponge-associated actinobacteria, have attracted increasing attention in recent years. Sun W. et al. explore the culturable actinobacterial diversity from sponges in the South China Sea that produce aromatic polyketides. The report by Mahmoud and Kalendar focuses on the diversity of coral-associated actinobacteria; the results may be helpful to understand how corals thrive under harsh environmental conditions. The inner tissue of higher plants is a special habitat. The article by Khieu et al. provides evidence that actinobacteria associated with medicinal plants have the potential to produce novel biological compounds. Finally, Trujillo et al. review the endophytic actinobacteria, in particular the interaction and environmental adaptations of *Micromonospora* co-occurring with plants.

We are delighted to present this research topic in Frontiers in Microbiology. We hope that this e-book will be interesting and useful to the readers of the journal and broaden the knowledge of actinobacteria in harsh environments. The information available above is promising but still limited. In the future, the application of innovative isolation and cultivation techniques, and –omics methods will undoubtedly unveil more unexpected and exciting aspects of actinobacteria in special and extreme habitats, and illuminate especially their ecophysiological function in nature.

## Author contributions

SQ organized this topic and wrote the editorial article. WL also wrote the editorial article. SD and WH are the co-editors of the topic and discussed the writing.

### Conflict of interest statement

The authors declare that the research was conducted in the absence of any commercial or financial relationships that could be construed as a potential conflict of interest.
